# A scorecard for assessing functionality of community health unit in Kenya

**DOI:** 10.11604/pamj.supp.2016.25.2.10524

**Published:** 2016-11-26

**Authors:** Duncan Ager, George Oele, Samuel Muhula, Susan Achieng, Moses Emalu, Mildred Nanjala, Sarah Kosgei, Susan Wanjiru, Peter Ofware, David Ojakaa, Meshack Ndirangu, Lennie Kyomuhangi

**Affiliations:** 1 Amref Health Africa in Kenya, Wilson Airport, off Langata Road, Nairobi, Kenya

**Keywords:** Scorecard, community health strategy, community health unit, functionality

## Abstract

**Introduction:**

In 2005, Kenya’s Ministry of Health (MOH) in its quest to improve health outcomes developed the Community Health Strategy (CHS) as a key approach. The MOH and partners grappled with the challenge of managing the functionality of the Community Health Units (CHUs). Amref Health Africa in Kenya developed a replicable CHUs Functionality Scorecard for measuring and managing the functionality of CHUs.

**Methods:**

We designed and piloted the CHU Functionality Scorecard at 114 CHUs in Rift valley province in Kenya. The scorecard categorized CHUs as Functional, Semi-functional, or Non-Functional. We used before and after design to assess the functionality of the CUs.

**Results:**

Over seven quarters (January 2012 to September 2013). The proportion of functional CHU increased from 3.5% to 82.9%, Semi-Functional reduced from 39% to 13% while Non-Functional reduced from 58% to 4%. The greatest improvements were noted in Community Health Volunteers (CHVs) receiving stipends, CHVs with referral booklets, monthly dialogue days, actions planning, chalk boards, and CHVs reporting rates.

**Conclusion:**

The CHU functionality scorecard is a valuable tool for the management of performance, resource allocation, and decision making. We recommend the adoption of the Functionality Scorecard by the Kenya Government for country-wide application. We recommend: further work in defining Advanced Functionality and incorporating the same into the scorecard; and implementation research on long term sustainability of CHUs.

## Introduction

Experiences in the last decade have demonstrated that in resource limited settings, health interventions that focus on building capacities at individual, household, and community levels for appropriate self-care, prevention, and care-seeking behavior are effective in improving maternal, newborn, and child health outcomes [1–3]. Such interventions have potential to address socio-cultural root causes of delays in decisions to seek skilled care from health facilities.

In the quest to improve access to equitable health services and health outcomes Kenya’s Ministry of Health developed the Community Health Strategy as a key approach [4, 5]. At its design, the Community Health Strategy included: establishing a Community Health Unit to serve a local population of 5,000 people; instituting a cadre of well trained Community Health Volunteers (CHVs) each providing services to 20 households; supporting every 25 CHVs with a Community Health Extension Worker (CHEW); and ensuring that the recruitment and management of the CHVs is carried out by Community Health Committees [6]. One of the strategic objectives for the health sector is to increase national coverage with the Community Health Strategy by strengthening and/or establishing 8000 Community Health Units across the country [7]. Since 2006, the Ministry of Health in Kenya has deployed the Community Health Strategy for delivery of an essential package of preventive and promotive health services at the community level [8]. Through this strategy, households and communities are empowered with skills to take an active role in health and health-related development by increasing their knowledge, skills and participation. The intention is to strengthen the capacity of communities to assess, analyze, plan, implement and manage health development initiatives thus effectively contribute to the country’s socio-economic development.

Through the Community Health Strategy with Functional Community Health Units, the Kenya’s health sector aims at enhancing community’s engagement in Health issues, access to health care in order to improve maternal, newborn, and child health (MNCH), improve individual productivity and thus reduce poverty, as well as enhance education performance [8]. The strategy outlines the following: types of preventive and promotive services to be provided by CHVs; skills levels of CHEWs required to deliver and support CHVs to deliver services minimum package of commodities required; and the management arrangements to be applied for effective operationalization of the strategy, including processes for enhancing linkages between health facilities and communities. The Community Health Strategy recognizes the pivotal role of the formal health system (dispensaries, health centers, and hospitals) in supporting community efforts through skills transfer, quality assurance of interventions, and support for referral processes.

By 2012, there was widespread establishment of Community Health Units in Kenya. The Ministry of Health and partners then grappled with the common challenge of how to measure and manage the functionality of the Community Health Units. This has been a problem across the country because Community Health Units were established without a common standard for moving them towards functionality - there had been no system or tool with agreed upon performance indicators. This has limited their ability to contribute to health outcomes even after significant costs of establishment have been invested. In response to this challenge, Amref Health Africa, itself having supported establishment of more than 700 Community Health Units across Kenya, made a decision to develop a Community Health Unit Functionality Scorecard for measuring and managing the functionality of multiple and geographically dispersed units both in Rural, Urban and North arid lands.

## Methods

This was a before and after study methodology which was looking at already formed community health units in terms of functionality. The scorecard categorized CHUs as Functional, Semi-functional, or Non-Functional. The sampling method was purposive for we took one of the projects with the highest number of community units in the Rural, Nomadic pastoralist, informal settlement in Rift valley province.

### Programme setting

In 2012, Amref Health Africa was supporting more than 700 Community Health Units geographically dispersed across the then eight provinces of Kenya (now 47 Counties). These CHUs were working with over 13,000 CHVs and close to 4000 members of CHCs, to deliver MNCH, HIV. Tuberculosis (TB), Water Sanitation and Hygiene (WASH) related health outcomes at the community level. The CHUs were supported through more than 36 projects of Amref Health Africa. They were spread across the rural, nomadic pastoralist, and urban informal settlement settings. Most of the Community Health Units had CHEWs, and all were linked to a local health facility. After its design, the Community Functionality Scorecard was piloted in one of the projects that was supporting 114 Community Health Units in the Rift Valley Province.

### Design of the community health unit functionality scorecard

Amref Health Africa developed a functionality scorecard with valid parameters and assessment tools aligned to the national community health strategy guidelines. Based on national guidelines and Amref Health Africa’s position on the role of CHVs, we operationally defined 17 functionality parameters required for a Community Health Unit to attain basic functionality (Table 1). We classified the parameters into inputs and outputs, and under outputs classified three as cardinal elements; we defined cardinal elements are those without which a Community Health Unit cannot be considered as functional even if it meets all other requirements, because of the pivotal role each of them plays in enabling the unit deliver health outcomes.

**Table 1:** Functionality parameters of a community health unit classified into inputs, outputs, and cardinal elements, and operational standards

	Functionality parameter		Operational standard
Inputs	Community health extension workers trained		Two extension workers trained per Community Health Unit
	Community health committee trained		Seven to 13 members of a community health committee trained using the national curriculum
	Community health volunteers trained		All community health volunteers trained on the basic package of the national curriculum
	CHVs provided with commodity kits		Each CHV provided with a portable bag with commodities and tools agreed upon with the sub-county health management team.
	Trained CHVs have MOH 513 and 514 reporting tools		All trained CHVs have MOH 513 and MOH 514 tools as part of their kit
	Community unit has a chalk board		The Community Health Unit has a chalk board (MOH 516) or an improvised one such as a blackboard displayed in a public place
	Trained CHVs have MOH 100 referral booklets		All trained CHVs have MOH 100 referral booklets and there is evidence they are using them.
	Transport mechanisms for use by the CHVs		The Community Health Unit has at least 10 functional bicycles are another appropriate mode of transportation for use by CHVs
	Support supervision		The sub-county health management team conducts data informed support supervision visit to the Community Health Unit at least every six months
	Output based stipends		CHVs reporting using MOH 514 tool receive standard/agreed upon stipend based on submission of a complete report each month.
Outputs	Action planning		The community health committee has a current written action plan for the Community Health Unit clearly stating the activities, planned dates of the activities, persons responsible, funds required, and sources of funds.
	Community health committee meetings		The community health committee meets each month and there are filed minutes
	CHV’s monthly meetings		The CHVs conduct monthly meetings to address needs and there are filed minutes
	Cardinal Elements	CHVs report monthly	At least 80% of CHVs in a Community Health Unit submitting a complete MOH 514 tool to the health extension worker each month.
		Dialogue days conducted	Community health committees leading quarterly dialogue days with CHVs and community members, and minutes of the meetings filed.
		Health action days	The Community Health Unit conducts monthly health action days based on the community health committee action plan and infirmed by data from the chalkboard (MOH 516)
	Sustainability initiative		The Community Health Unit have a livelihood strengthening initiative for CHVs

We further sequentially ordered the 17 parameters to represent the journey that a Community Health Unit follows from inception to basic functionality (Table 2); this was to enable rational decision making in investing resources, since fulfillment of certain parameters are pre-conditions for latter parameters i.e. there is a cause-effect relationship and interdependency among the elements of functionality.

**Table 2:** The 17 functionality elements of a community health unit organized sequentially to represent the journey that it follows from inception to maturity

1	CHEWs trained
2	CHC trained
3	CHVs trained
4	CHVs supplied with CHV kits
5	All trained CHVs have MoH 514
6	**CHV reporting rate above 80%**
7	CHU has a chalkboard
8	All trained CHVs have referral booklets
9	CHU action plan developed
10	Quarterly CHC Meeting held
11	CHVs monthly Meetings
12	All reporting CHVs (MoH 514) receiving stipend
13	**Monthly dialogue days held**
14	**Quarterly Health Action Days held**
15	DHMT supervisory visit conducted
16	CHU has bicycles for use by CHVs
17	CHU having a sustainable initiative(IGAs)

The scorecard articulates interdependency amongst the various Community Health Units’ structures and elements namely: the importance of a strong workforce and materials; motivation and performance management; comprehensive capacity enhancement of the work force; an enabling environment for all actors such as means of transport for CHVs and community health extension workers; importance of embracing sound processes in selection of community health committees for strong governance, and CHVs; health information systems; effective supportive supervision; and sustainability.

In order to translate data on the functionality elements into a score card, a score of one (1) is awarded when a criterion is met and zero (0) when it is not. The total score is calculated out of 17 and a percentage obtained for each Community Health Unit. Based on the percentage score obtained, a CHU is categorized as either Functional, Semi-functional, or Non-Functional (Table 3). Finally we translated the functionality parameters into a checklist (Table 4).

**Table 3:** Functionality categories and corresponding ranges of percentage scores

Functionality categories	Range of percentage (%) scores
Functional	>80% + All the three cardinal attained.
Semi-Functional	>50% to <80%
Non-Functional	<50%

**Table 4:** Sample data entry template - community health unit functionality assessment

County	Sub-County	Name of Community Health Unit	Name of Link Health Facility	Catchment population of Community Health Unit	CHEWs trained	CHC trained	CHVs trained	CHVs supplied with CHV kits	All trained CHVs have MoH 514	CHV reporting rate above 80%	CHU has a chalkboard	All trained CHVs have referral booklets	CHU action plan developed	Quarterly CHC Meeting held	CHVs monthly Meetings	All reporting CHVs (MoH 514) receiving stipend	Monthly dialogue days held	Quarterly Health Action Days held	DHMT supervisory visit conducted	CHU has bicycles for use by CHVs	CHU having a sustainable initiative(IGAs)	Score (total)	Score (%)	Functionality Status
Baringo	Mogotio	Emining	Emining Health Centre	7213	1	1	1	0	1	1	1	1	1	1	1	0	1	1	1	1	1	15	88	F
Baringo	Koibatek	Solian	Solian Dispensary	5524	1	1	1	0	1	1	0	0	1	1	1	0	1	1	1	0	1	12	71	SF
Baringo	Koibatek	Poror	Eldama Ravine District Hsp	5408	1	1	1	0	1	1	1	0	0	1	1	0	1	1	1	0	0	11	65	SF

^+^Cardinal elements of functionality are in bold

### Application of the community health unit functionality scorecard

We managed the application of the Functionality Scorecard through an eight steps process, working with and supporting the Ministry of Health counterparts and CHUs. The Ministry of Health Sub-County Health Management Team (SCHMT) took lead in the assessment process - from design of the assessment to analysis and interpretation of data, identification and prioritization of actions, and review of progress.

#### Step 1: conducted mapping to identify the community health units to be assessed

Working with the SCHMTs, we identified 114 Community Health Units that had been formed. We then developed a data entry template in micro-soft excel, allowing entry of information on the location of each Community Health Unit, the link health facility, the catchment population, and all the Functionality Parameters (Table 4).

#### Step 2: identified and orientated personnel on data collection

We oriented project officers, community health strategy focal persons, and research assistants as data collectors using the checklist covering all the elements of the functionality scorecard (Table 4). This orientation took one day. During pilot testing, it took approximately thirty minutes to complete the checklist.

#### Step 3: conduct functionality assessment of the selected community health units

The initial assessment was conducted between 30th April 2012 and 5th May 2012 and covered 114 Community Health Units. During a period of five days, the trained data collectors visited each of the CHUs. Respondents included CHVs, community health extension workers, and Community Health Committee members. CHVs were the respondents for the background information and service delivery; Community Health Extension Workers were respondents in performance enhancement elements and community based health information systems; and community health committee members were respondents in leadership and governance sections.

#### Step 4: data entry and analysis

Data was entered into an Epi info database and cleaned using the same program. The data was then transferred into a Micro-soft Excel spread sheet and presented in the form of a scorecard method. In the score card, presence of a particular parameter was depicted by figure one while absence of a parameter is equated to zero (Table 5). We conducted descriptive analysis and generated reports presented in tables and chart. In the actual scorecard, entries of figure one were shaded green, while entries of figure zero were shaded red to foster rapid identification of areas of weakness

**Table 5:** Template for functionality scorecard for each community health unit

County			
Sub-county			
Name of Community Health Unit:			
Link Health Facility:			
Catchment Population of Community Health Unir			
		**Yes**	**No**
**Inputs**			
Existence of trained CHEWs (2 per CU)			
Existence of trained CHC (7, 9, 11 or 13 based on population)			
Existence of trained CHVs (number of CHVs based on population density)			
CHVs provided with kit containing commodities agreed upon with the SCHMT or CHMT			
All trained CHVs have MOH 513 and MOH 514 tools			
Availability of a chalk board (MOH 516)			
All trained CHVs have referral booklets			
All reporting CHVs (using MOH 514) receiving monthly stipend of Ksh. 2000			
CHU has adequate means of transport (at least 10 bicycles) for use by CHVs			
Supervision of CU by SCHMT (at least once every six months)			
**Outputs**			
CHU has a plan of action (check wall or file)			
CHCs holding quarterly meetings (check minutes in file)			
CHVs holding monthly feedback meetings (check minutes in file)			
Existence of a sustainability initiative (discus with CHEW, CHC, & CHVs)			
Cardinal Elements for Basic Functionality			
CHV reporting rate above 80% in the CU			
Quarterly dialogues taking place (check reports from the file)			
Health Action Days taking place each month (check reports from the file)			
Total Score out of 17			
Percentage (%) Score			
**Functionality Categorization**			
Key	**Functionality Categorization**		
Yes - Fulfilled (Score one - 1)	≥80% - Functional+		F
No - Not fulfilled (Score zero - 0)	>50 to <80% - Semi Functional		SF
	≤50% - Non-Functional		NF

+ Note: The three (3) cardinal elements (15, 16, 17) MUST all be fulfilled for a CU with ≥80% score to be functional

#### Step 5: data dissemination and validation

We shared the data with Community Health Extension Workers, Community Health Volunteers, and Community Health Committees for verification and validation. Any errors or anomalies are corrected at this point.

#### Step 6: reporting

We prepared summary reports for each Community Health Units (see template used for this in (Table 3) and an overall report to the Sub-County Health Management Team for use.

#### Step 7: action planning

The Community Health Extension Workers and Community Health Committees in each CHU provide leadership for dialogue on the report and preparation of plan of action for improvement with technical support from the respective Sub-County Health Management Team.

#### Step 8: monitoring and evaluation

The sub-county community strategy focal person is the custodian of the database. Assessment on functionality is done quarterly and the Scorecard updated to track performance of each community health unit.

## Results

### Demographic and institutional factors associated with health facility delivery

**Figure 1:**
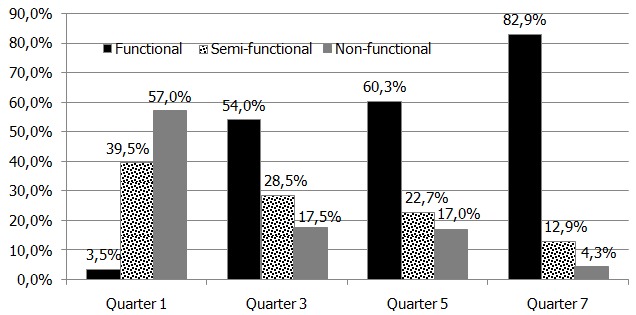
Changes in functionality status of community health units over a period of seven quarters

We observed marked improvement in the functionality of targeted Community Health Units as a result of application of the scorecard over a period of seven quarters (January 2012 to September 2013) using the definition of the parameters there was a uniform understanding of the formation and management of the Community Health units in the province, sharing of the scorecard every quarter brought competition among the CHEWs, CHCs and CHVs between different units with end results in improved engagement of the Community on health issues, the report rates moved from 40% to 80% actually it doubled. The cardinal parameters became the measure of performance (Figure 1). During this period, the proportion of functional Community Health Units increased from 3.5% (4 out of 114) to 82.9% (116 out of 141). The tool could easily be used to assess functionality of all community health units whether in rural, rural-urban, nomadic or urban areas without difficulties. The greatest improvements were noted in CHVs receiving stipends, CHVs with referral booklets, monthly dialogue days, actions planning, chalk boards, and CHV reporting rates (Table 6).

**Table 6:** Comparison of scores on community health units functionality elements between the first and seventh quarter

	Functionality Parameter	Quarter 1		Quarter 7		
		Number (n=114)	%Score	Number (n=140)	% Score	Percentage change
1	CHEWS trained	112	98%	135	96%	-2%
2	CHC trained	63	55%	137	98%	43%
3	CHVs trained	75	66%	133	95%	29%
4	CHVs supplied with CHV kit	9	8%	51	36%	29%
5	All trained CHVs have MoH 413, 514	67	59%	135	96%	38%
6	CHU has a chalk board (MoH 516)	53	46%	127	91%	44%
7	All trained CHVs have referral booklets	18	16%	130	93%	77%
8	CHU action plan developed	50	44%	132	94%	50%
9	Quarterly CHC meeting held	62	54%	127	91%	36%
10	CHVs monthly meeting held	63	55%	130	93%	38%
11	Reporting CHV receive stipend	0	0%	129	92%	92%
12	Monthly dialogue days held	46	40%	132	94%	54%
13	Quarterly health action days held	40	35%	109	78%	43%
14	DHMT supervisory visit conducted	57	50%	117	84%	34%
15	CHU has bicycle for use by CHVs	31	27%	70	50%	23%
16	CHU has a sustainability initiative (IGA)	23	20%	58	41%	21%
17	CHV reporting rate above 80%	42	37%	113	81%	44%

## Discussion

The results show that the Functionality Scorecard as an effective tool for managing Community Health Units to achieve basic functionality thus laying the foundation for them to deliver health outcomes. The application of the scorecard led to marked improvement in 16 elements of functionality, with marked changes in CHVs with referral booklets, Community Health Units holding monthly dialogue days and action days guided by evidence based actions plans, and CHV reporting rates. Notably these had been the weakest elements at the beginning of the application of the scorecard. Supporting Community Health Units to ensure they have tools, are conducting dialogue days and action days, reporting and using the data for local decision making are crucial steps in enabling them deliver value in terms of health outcomes. Although we did not use the Basic Functionality Scorecard to assess health outcomes, data from several Community Health Units managed by Amref Health Africa Scorecard has indicated marked improvements in health outcomes. For example, in one of its programs in Makueni County which is using the CHUs adopted the scorecard and they noticed that the skilled attended delivery improved from 37.5% to 44.2 % in 12 months, and newborn deaths declined to zero from four in the previous year. These findings are consistent with other findings of Amref Health Africa with regards to the effectiveness of the Community Health Strategy in delivering health outcomes especially related to maternal and child health outcomes [3].

Amref Health Africa is now working to improve the Functionality Scorecard so that after a Community Health Unit has attained basic functionality, effort shifts to moving it towards advanced functionality. The primary principle of the Community Health Unit Functionality Scorecard is to inform and influence decision making among stakeholders involved in the management of Community Health Units. As evidenced in this paper, Amref Health Africa has used the tool to manage progression of Community Health Units towards basic functionality and now moving them towards advanced functionality. Sub-county health management teams and project teams are using the scorecard using the eight steps process described under results, enabling them make the following decisions and act: gather baseline data on functionality and set benchmarks to track performance of Community Health Units; plan and set priority actions for specific Community Health Units, ensuring that investments in each unit address the weak or missing elements and in a logical order; equity in resource allocation between different Community Health Units, as well as between different sub-counties, since allocation is based on needs - for example, the sub-county health management team is able to direct implementing partners to address priority needs within existing units; rapidly identify Community Health Units that can be moved from basic functionality to advanced functionality through provision of key technical skills; provide performance based incentives to CHVs using a fair and objective platform to guide provision of performance based incentives to CHVs. Application of the Functionality Scorecard has emerged as a motivation to CHVs, Community Health Committees, and Community Health Extension Workers since the teams are able to clearly assess and validate their performance.

## Conclusion

The community health unit functionality scorecard is a valuable tool for the management of performance, resource allocation, and decision making for multiple and geographically dispersed community health units. The scorecard can be used by health projects that use the community health strategy as a service delivery platform to improve health outcomes at scale. We recommend the adoption of the Functionality Scorecard by the Kenya Government for country-wide application. We recommend further work in: defining advanced functionality and incorporating the same into the scorecard; and implementation research on long term sustainability of community health units.

### What is known about this topic

- The government of Kenya is working on formally rolling out standards for measuring functionality of community units to track their performance. We have recommended this scorecard to the government for consideration;
- There still exist glaring gaps in implementation of the community strategy in Kenya with noticeable disparities in functionality of community units across the country. In spite of this, communities appreciate the community strategy and its contribution to improved health status in Kenya;
- Kenya currently has about 2,500 CUs and is in the process of establishing additional 8,000 CUs by 2017.

### What this study adds

- The scorecard for measuring the community health unit functionality is the first of its kind in Kenya. The tool is simple and user friendly;
- The tool is instrumental for the guidance on what one needs to have to initiate a community health unit since the score card parameters are also the steps in initiating a unit from what needs to be done first to the last;
- The score card is a management tool to help assess the performance, resource allocation and decision making. It is able to provide guidance on budgeting for a CHU.

## Abbreviations

CHEW: Community Health Extension Worker
CHV: Community Health Worker
MNCH: Maternal, Newborn, and Child Health
CHC: Community Health Committee
MOH: Ministry of Health
RH: Reproductive Health
TB: Tuberculosis
PMTCT: Prevention of Mother to Child Transmission of HIV
WASH: water sanitation and hygiene
